# Quantitative magnetic resonance imaging to aid clinical decision making in autoimmune hepatitis

**DOI:** 10.1016/j.eclinm.2022.101325

**Published:** 2022-03-21

**Authors:** Michael A. Heneghan, Elizabeth Shumbayawonda, Andrea Dennis, Refah Z. Ahmed, Mussarat N. Rahim, Michael Ney, Loren Smith, Matt Kelly, Rajarshi Banerjee, Emma L. Culver

**Affiliations:** a Institute of Liver Studies, King's College Hospital, NHS Foundation Trust, Denmark Hill, London, United Kingdom; b Perspectum Ltd, Oxford, United Kingdom; c John Radcliffe Hospital, Oxford University Hospitals NHS Foundation Trust, Oxford, United Kingdom

**Keywords:** Remission, Fibro-inflammation, Non-invasive imaging, Stratification, cT1

## Abstract

**Background:**

In autoimmune hepatitis (AIH), clinical practice and treatment guidelines frequently diverge as a reflection of disease heterogeneity and challenges in achieving standardised care. We sought to explore the utility of multiparametric (mp) MR in patients with AIH, and the impact of this technology on physicians’ decision making and intended patient management.

**Methods:**

82 AIH patients, recruited from two sites between June and November 2019 as part of an observational cohort study, underwent non-contrast MRI alongside their standard clinical investigations. Correlations between iron-corrected T1 (cT1) and other markers of disease were investigated alongside the utility of imaging markers to risk stratify patients in biochemical remission. The impact of mpMR on clinical decision making was evaluated using pairwise t-tests. The discriminatory ability of the imaging markers was assessed using area under the receiver operating characteristic curves (AUCs).

**Findings:**

cT1 had a significant impact on clinician intended patient management (*p*<0.0001). cT1 correlated with ALT (*p* = 0.0005), AST (*p*<0.001), IgG (*p* = 0.0005), and liver stiffness (*p*<0.0001). Patients in deep biochemical remission (*N* = 11; AST/ALT <50% upper limit of normal [ULN] and IgG <12 g/L) had low cT1, while 7/34 in normal biochemical remission (AST/ALT between 50 and 100% of ULN) had high cT1 and were at risk of disease flare. cT1 measures of disease heterogeneity, ALP and bilirubin made the best predictor of those not in biochemical remission (AUC:0.85).

**Interpretation:**

This study investigates the impact of mpMR results on intended clinical management in a real world setting. Findings showed that mpMR demonstrated a significant impact on clinical management of AIH and has the potential to inform patient risk stratification.

**Funding:**

This paper presents independent research supported by the Innovate UK grant (104,915).

**Research in context**

**Evidence before this study**

We searched PubMed, MEDLINE, Embase and Google Scholar from 2003 to 2020. Search terms included autoimmune hepatitis [MeSH Terms], “autoimmune hepatitis”, “autoimmune hepatitis guidelines”, “noninvasive imaging markers in autoimmune hepatitis”, and “corrected T1 in autoimmune hepatitis”. We observed that although clinical management relies on needle biopsy as a gold standard (despite its limitations and inappropriateness for long-term monitoring), there is a need for more prognostic non-invasive biomarkers which predict the risk of treatment failure, relapse, or disease progression to support clinicians and individualize management strategies.

**Added value of this study**

This study presents a comparison of the utility of multiparametric magnetic resonance (MR) biomarkers to other commonly used non-invasive markers to identify patients in biochemical remission with previously undetected active sub-clinical disease at high risk of disease relapse. MR biomarkers performed comparatively better to discriminate between those in biochemical remission vs active disease as well as identifying those in biochemical remission with undetected active disease at risk of relapse. Multiparametric MR biomarkers also showed significant impact on physician's intended clinical management plans for patients with autoimmune hepatitis.

**Implications of all the available evidence**

This study demonstrates the potential of multiparametric MR biomarkers as tools for improved patient management and for early detection of at risk patients most likely to relapse due to active sub-clinical disease. By also characterising the heterogeneity across the liver, these biomarkers have the potential to provide additional benefit compared to using serum biochemistry and liver stiffness alone.

Alt-text: Unlabelled box

## Introduction

Autoimmune hepatitis (AIH) is a challenging condition that presents in both acute and chronic forms in patients of all ages.^1^ It remains a diagnosis of exclusion since there is no disease-specific test and one third of patients present with advanced liver disease. Corticosteroids and non-selective immunosuppression are currently the mainstay of treatment. Disease relapse is common and affects up to 80% of patients after treatment withdrawal. Moreover, undesired corticosteroid-related side effects are considerable,^2^ and up to 50% of patients can develop cirrhosis despite therapeutic intervention.^3^ AIH has a female predominance, with the majority of most patients requiring life-long monitoring.^1^^,^^3^

To-date, liver biopsy is considered essential for diagnosis of AIH in accordance with all clinical guidelines, and can highlight co-existence with non-alcoholic steatohepatitis, viral hepatitis, and variant syndromes.^4^^,^^5^ It is also useful for evaluation of treatment response and to guide therapy by quantification of hepatic inflammation and staging of liver fibrosis.

Monitoring of liver inflammation in AIH relies on non-invasive assessment such as liver biochemistry (alanine transaminase (ALT) activity) and immunoglobulin G (IgG) levels. Liver biochemistry can be insensitive to changes in fibro-inflammation in the liver and one cannot exclude underlying residual active hepatic inflammation in the presence of ‘normal LFTs’ (liver function tests).^6^ Some clinical guidelines recommend repeat “on-treatment” histological assessment to confirm complete resolution of histological inflammation to aid in long-term therapeutic management considerations.^4^, ^5^, ^6^ This has not been universally adopted.^7^ In practice, liver biopsy is performed at diagnosis, for an unexplained and persistent flare in liver biochemistry/IgG and when considering treatment cessation or withdrawal.^5^ Liver biopsy however is invasive and not liked by patients. This paucity in follow-up histological assessment has resulted in a need for objective, quantitative, reproducible, and accurate non-invasive methods of assessing liver health in this population.^5^

Serum-based biomarker panels, such as FibroTest®,^8^ serum AST/platelet ratio index (APRI),^9^ Fibrosis-4 index (FIB-4)^10^ and the enhanced liver fibrosis (ELF) test^11^ have been used anecdotally in patients with AIH; their use requires further validation.^5^ Non-invasive imaging techniques, including vibration-controlled transient elastography (VCTE; Fibroscan®, Echosens, France), magnetic resonance elastography (MRE) and acoustic radiation force impulse imaging (ARFI) are confounded by hepatic inflammation.^5^ VCTE has a high coefficient of variation although is often used after 6-months of treatment to monitor disease response and progression of fibrosis.

Multiparametric magnetic resonance (mpMR) techniques have been able to impact clinical practice in medical fields such as oncology (prostate and breast cancer) and cardiology, due to the generation of quantitative biomarkers.^12^, ^13^, ^14^ mpMR has also demonstrated its utility in clinical pathways for the assessment of liver disease.^15^, ^16^, ^17^, ^18^ Iron corrected T1 (cT1) from Liver*MultiScan* (Perspectum, Oxford) is an objective mpMR measurement of fibro-inflammation with low inter-observer variability and high repeatability over time,^19^^,^^20^ as well as a very low coefficient of variation in monitoring liver disease at multiple timepoints.^20^ cT1 correlates with histology^16^^,^^21^, ^22^, ^23^ and has been shown to detect early responses to treatment in non-alcoholic fatty liver disease.^24^ More recently, cT1 has also shown utility in monitoring disease regression and has correlated with histological fibrosis and inflammation in AIH patients in biochemical remission.^25^ Importantly, cT1 outperformed other surrogate markers (VCTE-liver stiffness and elevated liver enzymes) to predict future disease flare in AIH patients, with a cT1 ≥800 ms associated with *a* ≥ 20% chance of experiencing a future disease flare, rising to ≥75% chance with a cT1 ≥1000 ms.^25^

The aim of this study was to understand the impact of cT1 on a physician's intended management plan for patients with AIH. Our objective was to investigate the effect of mpMR results on a physician's decision making and subsequent intended patient management plans for patient with suspected or confirmed AIH compared with usual standard of care.

## Methods

### Patient recruitment

82 adult patients (*N* = 59 from King's College Hospital NHS Foundation Trust and *N* = 23 from Oxford University Hospitals NHS Trust) with an established biopsy-proven diagnosis of AIH were prospectively recruited into this study between June 2019 and November 2019. Exclusion criteria included: (1) inability or unwillingness to give consent, (2) those with contraindications to the MR procedure (such as pregnancy or non-MR compatible implants), (3) those with any clinical doubt to their underlying aetiology of liver disease, or (4) patients with evidence of current overt hepatic decompensation (such as encephalopathy, gross ascites, or variceal haemorrhage).

### Diagnostic criteria

All patients recruited in this study had biopsy confirmed AIH (diagnosed 3.1 years (range: 0–14.4 years) before recruitment). Moreover, at the time of diagnosis, all patients fulfilled the revised,^26^ and simplified International Autoimmune Hepatitis Group (IAIHG)^27^ criteria with all patients being classed as having probable or definite AIH (score of ≥6).

### Ethics statement and registration

Patients gave written informed consent to participate in the study. Local ethical approval was gained via the National Research Ethics Service, West Midlands (Black Country, reference 19/WM/0111), along with appropriate data sharing, confidentiality, and collaboration agreements. The study was registered as a clinical investigation (NCT03979053). Principals of Good Clinical Practice and those of the 1975 Declaration of Helsinki were observed. All patient-identifiable information was kept securely and encrypted within the servers at the study sites.

### Study procedures

**Figure 1:**
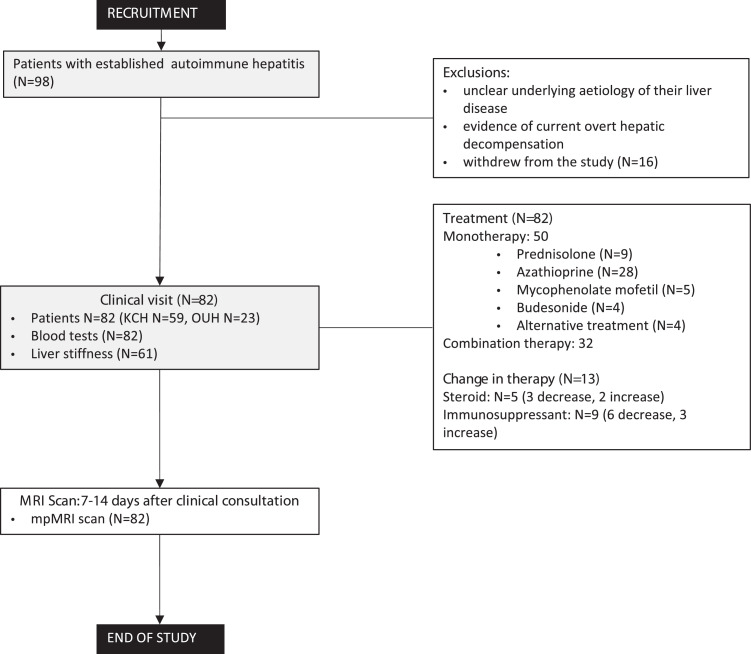
The identification, recruitment, and active study procedure followed in this project.

Patients underwent non-invasive assessment including clinical history, examination, blood panel analysis, VCTE liver stiffness (LS; Fibroscan®, Echosens, France) and non-contrast mpMR. Figure 1 shows a summary of the protocol followed in this study.

### MpMR protocol

The mpMR scanning protocol was installed, calibrated and phantom tested on a 3 T Siemens Prisma MRI scanner (Siemens Healthcare GMBH, Erlangen, Germany) at the Oxford Centre for Magnetic Resonance (OCMR) and a 3 T Siemens Skyra MRI scanner (Siemens Healthcare GMBH, Erlangen, Germany) at Alliance Medical, London. Four single transverse slices were captured throughout the liver centred at the porta hepatis. Anonymised MR data were analysed off-site using LiverMultiScan® software (Perspectum Ltd., United Kingdom) by specialised imaging analysts trained in abdominal anatomy and artefact detection. cT1 maps of the liver were delineated into whole liver segmentation maps using a semi-automatic method, as extensively described by Bachtiar and colleagues,^19^ and expressed as the median value within the map. cT1 interquartile range (IQR), a measure of the spread of cT1 values across the liver, and the count (expressed as a percentage) of the pixels in the liver map above a pre-defined threshold of 800 ms (pcT1), both of which represent disease heterogeneity, were also extracted from the whole liver segmentation maps. The mpMR analysis was completed by analysts blinded to the clinical data.

### Assessment of clinical impact of cT1

To address the primary objective, clinicians were asked to score the utility of the technology at two stages. The first score, which reflected the anticipated benefit of mpMR, was given immediately after the clinic consultation once the clinician had reviewed the existing patient information and prior to the patient attending the MRI scan. After receiving the mpMR report, the clinicians were asked to provide a second score reflecting the impact of mpMR on the clinical management plan for the patient. For both scores and for each individual patient, a 10-point Likert scale was used to record the clinicians’ level of agreement or disagreement concerning the utility of the technology. On the Likert scale used, a score of 1 indicated no confidence in the utility of LiverMultiScan, whilst a score of 10 indicated complete confidence.

### Definitions of disease inactivity and biochemical remission

**Figure 2:**
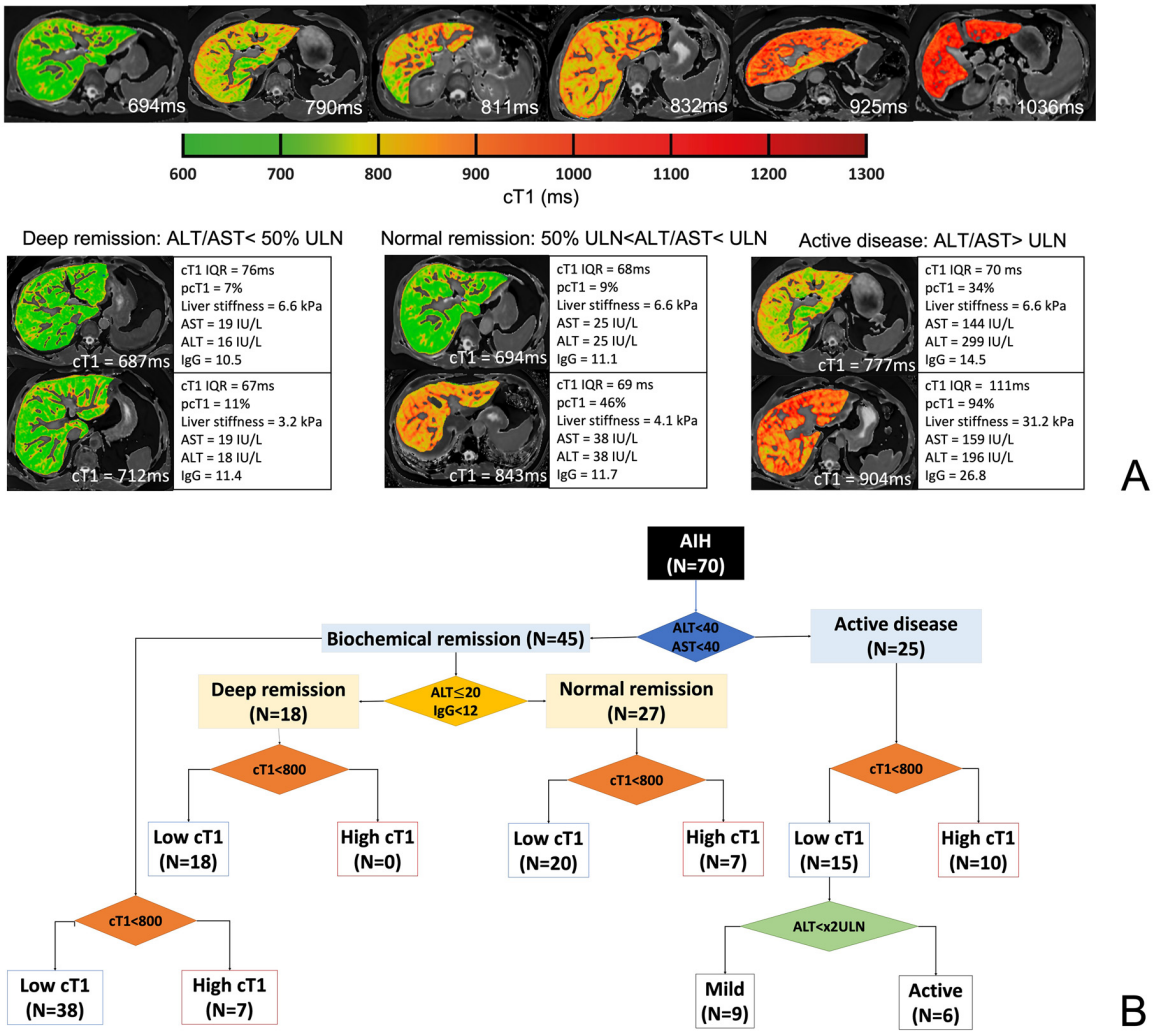
(A) Changes in cT1 (ms) associated with increasing fibro-inflammatory burden and differences in cT1 between remission groups. In the images, lower values (cooler colours in maps and colour bar) represent areas with lower cT1 values and therefore lower fibro-inflammation, while higher cT1 values (warmer colours) represent areas of the liver with higher fibro-inflammation. (B) Classification of patients using biochemistry and cT1 to identify the spread of patients with resolved biochemistry that still have active fibro-inflammation.

Clinical guidelines define one of the goals of treatment in AIH to be complete biochemical remission.^4^^,^^5^ As ALT remission has been shown to correlate with outcomes,^5^ patients in this study were classified into two groups according to biochemistry. Biochemical remission groups were formed around the upper limit of normal (ULN) of aspartate aminotransferase (AST) and ALT^4^ with those in biochemical remission having AST and ALT less than the ULN (i.e. AST≤40IU/L and ALT≤40IU/L), while those not in biochemical remission were classified as having active disease. Similar to work by Hartl and colleagues,^28^ to further understand the utility of mpMR to identify the existence of gradations in biochemical remission and to identify those with active sub-clinical disease, patients in biochemical remission were further classified as being either in deep (ALT ≤50% ULN (ALT≤20IU/L) and IgG<12 g/L) or normal biochemical remission. Lastly, as a cT1 of >800 ms has previously been shown to relate to increased risk of disease relapse,^25^ and is indicative of continued active fibro-inflammatory disease^29^ it was used as a threshold to identify those with active sub-clinical disease (Figure 2).

### Statistics

Descriptive statistics were used to summarise baseline participant characteristics. Continuous variables were reported as mean and standard deviation and categorical variables were reported as frequency and percentage. The time between diagnosis biopsy and recruitment into the study (disease duration) was also calculated and reported as median and inter-quartile range. For the primary objective, paired t-tests were used to assess the impact of cT1 on clinical decision making by compare the scores given by the clinicians before and after reviewing the mpMR results.

To assess the agreement between non-invasive and biochemical biomarkers, correlations between all markers (median cT1 (cT1), cT1 IQR, pcT1, and LS) with blood tests were analysed using Spearman's correlation coefficient (r_s_). Mean differences between those in biochemical remission and those with active disease were compared using the Kruskal Wallis test. Further to this, mean differences between those with low (cT1<800 ms) vs high cT1 (within each biochemical remission group respectively) were evaluated with the Kruskal Wallis tests.

The ability of cT1 to discriminate between those in biochemical remission from those not in biochemical remission were estimated using the area under the receiver operating characteristic curves (AUCs). Sensitivity, specificity, negative and positive predicting values (NPV, PPV respectively) for the predefined cT1 cut-off of 800 ms was calculated, with Youden's Index used to obtain the best cut-off and related sensitivity, specificity, NPV and PPV for pcT1 and liver stiffness. For the exploratory investigations into possible gradations in biochemical remission, Kruskal Wallis test tests were used to evaluate if any differences existed between those in deep biochemical remission and those in normal biochemical remission.

Descriptive statistics and correlations were performed using all patient's data, however, subsequent investigations after classification into biochemical remission groups were performed in the patients with both AST and ALT data available for analysis (*N* = 70). All statistical analyses were performed using R version 3.5.3 (R Core Team, Vienna, Austria), and values of *p*<0.05 were considered statistically significant. The Shapiro-Wilk test was used to assess normality of the metrics investigated.

### Sample size calculation

The sample size estimation was based on the primary objective and endpoint to assess the effect of MRI results on a physician's decision making and the number of patients in whom intended clinical management is changed when the MRI results are delivered. According to preliminary data from Arndtz and colleagues subsequently published,^25^ up to 50% of low-risk patients and up to 23% of high risk patients (as identified by blood serum markers) go on to develop a flare within next 12 months. Thus, in a hypothetical scenario, in a 100 patient cohort where 50 patients are considered to be low risk and 50 are considered to be high-risk, 25 low-risk patients will be incorrectly assigned low risk status (instead of high-risk) and will go on to develop a flare. The study also showed that mpMR can benefit up to 36% of patients and thus result in a change in management. Considering the null hypothesis that a clinician can correctly identify patient status (high/low-risk) 25–30% of the time and the worst case scenario where mpMR can benefit 15% patients (small effect size of 0.31–0.34), approximately 60 patients (assuming 80% power, alpha 0.05 and loss rate of 10%) would be required to test our hypothesis, with approximately 80 patients required if a loss rate of 25% is considered.

### Role of the funding source

The funding source had no role in the study design, data collection, data analyses, data interpretation, writing of the report and the decision to submit the manuscript for publication. All authors (MAH, ES, AD, RZA, MNR, MN, LS, MK, RB, ELC) has access to the data and jointly took the decision to submit the paper for publication.

## Results

### Description of cohort

All patients, median age of 52 years (range: 21–81), were consented and recruited over three months between June and October 2019 (Figure 1, Table 1). Seventy-seven percent were female. All participants were on either monotherapy or a combination of therapies at the time of recruitment. The time between the most recent liver biopsy and MRI scan (disease duration) was 23.1 months (IQR:46.7)​ months.

**Table 1:** Population demographics showing patient characteristics, blood panel and non-invasive liver assessment results. Statistical differences between those in biochemical remission (AST≤40IU/L and ALT≤40IU/L) and those with active disease have also been indicated. P-values for ALT and AST have not been added as these were used to define the biochemical remission groups.

	Whole cohort	Biochemical Remission		Active disease	p-value	Normal limits
		Deep	Normal			
Patient characteristics						
Cohort size	82	18	27	25		
Age	52±16	60±13	52±14	45±17	0.058	
Body Mass Index (kg/m^2^)	27.4 ± 5.6	26.0 ± 5.6	28.2 ± 4.7	26.8 ± 5.0	0.751	18.5–24.9
Serum Liver and Liver function tests						
Platelets (10^9/L)	222±91	257±78	228±98	205±89	0.196	
ALP (IU/L)	73±30	69±28	69±26	82±34	0.128	30–130
GGT (IU/L)	55±58	26±13	54±48	74±77	0.196	15–40
ALT (IU/L)	40±45	15±3	25±8	81±63	<0.001	10–45
AST (IU/L)	41±32	23±4	28±7	70±42	<0.001	15–42
Albumin (g/L)	43±4	45±3	43±4	42±4	0.125	32–50
Bilirubin (µmol/L)	14±8	12±5	14±10	16±8	0.114	0–21
Total serum globulins (g/L)	28±5	25±2	28±5	30±6	0.065	20–35
IgG (g/L)	13.1 ± 4.6	9.9 ± 1.5	13.3 ± 4.7	14.9 ± 5.0	**0.01**	6.5–18.5
Surrogate markers of liver health						
Liver stiffness measure (kPa)	9.3 ± 8.5	5.8 ± 3.8	6.7 ± 5.5	12.5 ± 10.9	**0.003**	
Fat (%)	4.1 ± 4.2	3.5 ± 2.0	4.0 ± 3.2	3.7 ± 4.6	0.289	100–400
cT1 (ms)	781±105	731±29	766±68	818±95	**0.002**	633–794
pcT1 (%)	35±27	18±11	32±26	48±30	**0.002**	

### Clinical assessment

All patients were assessed face-to-face in outpatient clinic. 69/82 (84%) were felt to have stable disease based on clinical parameters, liver biochemistry (+/-immunoglobulin levels), and continuation of treatment regimen. Thirteen out of 82 patients (16%) required a change in medication due to various reasons, including increased/reduced disease activity, planned escalation/de-escalation of therapy, intolerance of medication, or patient choice. 5/13 (6%) had their steroid treatment adjusted (3 decrease, 2 increase) and 9/13 (11%) had a change in their immunosuppressive therapy (6 decrease, 3 increase) (Figure 1).

### Surrogate imaging biomarkers and serum markers of AIH activity

cT1 correlated significantly with serum markers (ALT (r_s_=0.31, *p* = 0.005), AST (r_s_=0.47, *p*<0.001), IgG (r_s_=0.41, *p* = 0.001), GGT (gamma-glutamyl transferase; r_s_=0.49, *p*<0.001) and albumin (r_s_= −0.32, *p* = 0.036)), as well as with liver stiffness (r_s_=0.51, *p*<0.001), and disease duration (r_s_=0.32, *p* = 0.003). Furthermore, there were significant correlations between pcT1 and serum ALT (r_s_=0.33, *p* = 0.003), AST (r_s_=0.51, *p*<0.001), IgG (r_s_=0.35, *p* = 0.003), GGT (r_s_=0.5, *p*<0.001) and liver stiffness (r_s_=0.54, *p*<0.001) (Table 2).

**Table 2:** Correlations (R) between cT1 (ms) with serum liver function test results and other surrogate markers of liver health. All significant associations are highlighted in bold.

	cT1 (ms)	cT1 IQR (ms)	pcT1 (%)	Liver stiffness (kPA)
Serum Liver function tests (*N* = 82)				
ALT (IU/L)	0.31 (*p* = 0.005)	0.13 (*p* = 0.26)	0.33 (*p* = 0.003)	0.36 (*p* = 0.005)
AST (IU/L)	0.47 (*p*<0.001)	0.29 (*p* = 0.015)	0.51 (*p*<0.001)	0.48 (*p*<0.001)
Albumin (g/L)	−0.32 (*p* = 0.004)	−0.30 (*p* = 0.006)	−0.31 (*p* = 0.005)	−0.30 (*p* = 0.019)
ALP (IU/L)	0.07 (*p* = 0.51)	0.11 (*p* = 0.32)	0.09 (*p* = 0.43)	0.36 (*p* = 0.003)
Bilirubin (µmol/L)	0.07 (*p* = 0.51)	0.33 (*p* = 0.003)	0.06 (*p* = 0.58)	0.18 (*p* = 0.18)
GGT (IU/L)	0.49 (*p*<0.001)	0.22 (*p* = 0.075)	0.50 (*p*<0.001)	0.41 (*p* = 0.005)
IgG (g/L)	0.41 (*p* = 0.001)	0.18 (*p* = 0.15)	0.35 (*p* = 0.0031)	0.05 (*p* = 0.71)
Platelets (10^9/L)	−0.13 (*p* = 0.26)	−0.43 (*p*<0.001)	−0.14 (*p* = 0.21)	−0.30 (*p* = 0.018)
Total serum globulins (g/L)	0.50 (*p* = 0.0001)	0.22 (*p* = 0.10)	0.45 (*p* = 0.0004)	0.34 (*p* = 0.025)
Surrogate markers				
Liver stiffness (kPA; *N* = 80)	0.51 (*p*<0.001)	0.47 (*p*<0.001)	0.54 (*p*<0.001)	
Disease duration (years)	0.32 (*p* = 0.003)	0.15 (*p* = 0.17)	0.34 (*p* = 0.002)	0.20 (*p* = 0.13)

### Non-invasive imaging and disease activity

#### Active disease

**Figure 3:**
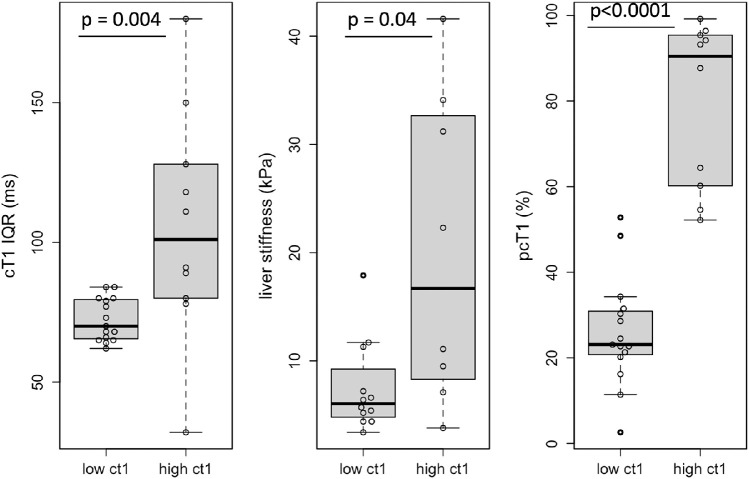
Markers that were significantly different between those with mild active disease (ALT < x2 ULN) vs those with active disease. Those with mild active disease (ALT < x2 ULN) and low fibro-inflammatory activity (cT1<800 ms) had significantly lower heterogeneity (cT1 IQR: *p* = 0.004; pcT1: *p*<0.0001) and liver stiffness (*p* = 0.04) compared to those with active disease (ALT > x2 ULN) and high cT1 (cT1>800 ms). The whiskers of the boxplots represent the minimum and maximum values, the box covers the first and third quartiles with a line indicating the median. All outliers falling outside the area covered by the whiskers are indicated. cT1 IQR (ms): cT1 interquartile range a measure of disease heterogeneity, liver stiffness (kPa) measured of fibrosis by transient elastography, pcT1 (%): the percentage of the pixels in the cT1 map above 800 ms, measure of disease burden and heterogeneity.

25 patients had active disease with 15 having cT1<800 ms. Of these 15, 60% had ALT less than x2 ULN (ALT: 47.9 ± 12.3 IU/L) and according to clinical guidelines^4^ have mild disease (Figure 2). The patients with mild disease had significantly lower cT1 IQR (*p* = 0.004), liver stiffness (*p* = 0.04) and pcT1 (*p*<0.0001) compared to those with cT1>800 ms (Figure 3).

#### Biochemical remission vs active disease

**Figure 4:**
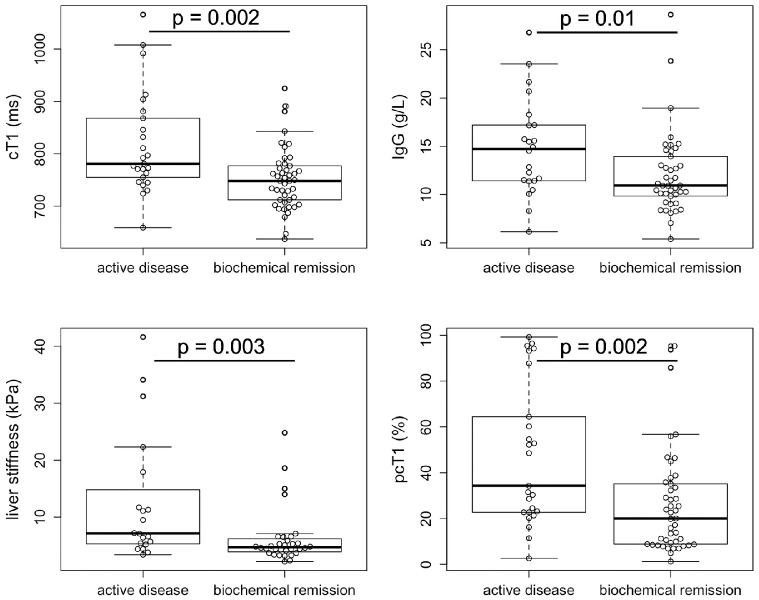
Markers that were significantly different between those with active disease compared to those in biochemical remission (AST≤40IU/L and ALT≤40IU/L). Those with active disease had significantly higher cT1 (*p* = 0.002), pcT1 (*p* = 0.002), liver stiffness (*p* = 0.003) and IgG (*p* = 0.01) compared to those in biochemical remission. The whiskers of the boxplots represent the minimum and maximum values, the box covers the first and third quartiles with a line indicating the median. All outliers falling outside the area covered by the whiskers are indicated.

Those with active disease had significantly higher cT1 (*p* = 0.002), IgG (*p* = 0.01), liver stiffness (*p* = 0.003) and pcT1 (*p* = 0.002) compared to those in biochemical remission (Figure 4). In the discrimination between biochemical remission and active disease, a cT1 of 800 ms had AUC:0.77 (95% CI: 0.63–0.9), 40% sensitivity, 84% specificity, 67% PPV and 70% NPV. In addition to this, a pcT1 of 48% had AUC:0.76 (95% CI: 0.62–0.9), 50% sensitivity, 94% specificity, 83% PPV and 75% NPV, while a liver stiffness of 5.4 kPa was found to have AUC:0.74 (95% CI: 0), 75% sensitivity, 69% specificity, 60% PPV and 81% NPV to discriminate between the two biochemical remission groups.

#### Biochemical remission and active sub-clinical disease

**Figure 5:**
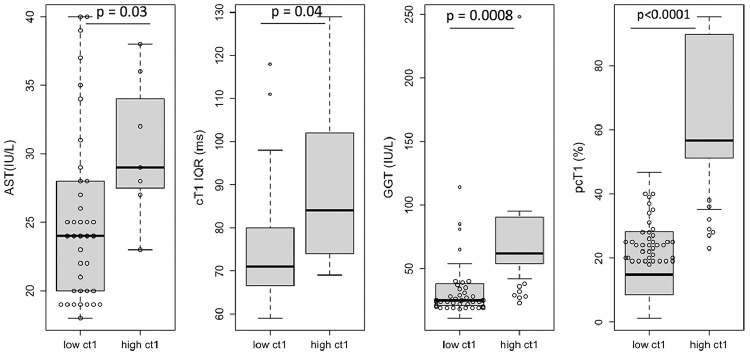
Markers that were significantly different between those in biochemical remission (AST≤40IU/L and ALT≤40IU/L) with (cT1<800 ms) vs without (cT1>800 ms) sub-clinically active disease on imaging. Those with clinically active disease on imaging had higher AST (*p* = 0.03), GGT (*p* = 0.0008), cT1 (*p* = 0.04) and pcT1 (*p*<0.0001) compared to those without clinically active disease on imaging. The whiskers of the boxplots represent the minimum and maximum values, the box covers the first and third quartiles with a line indicating the median. All outliers falling outside the area covered by the whiskers are indicated.

Of the 45 patients in biochemical remission, 18 were in deep biochemical remission and 27 were in normal biochemical remission (Figure 2). The 7 patients in normal biochemical remission with cT1>800 ms had significantly higher pcT1 (*p* = 0.0003) and GGT (*p* = 0.004) when compared to those in normal biochemical remission with cT1<800 ms. Moreover, when compared on a global scale with all other patients in biochemical remission, these 7 patients were found to have significantly higher AST (*p* = 0.03), cT1 IQR (*p* = 0.04), GGT (*p* = 0.0008) and pcT1 (*p*<0.0001) (Figure 5).

### Impact of cT1 on clinical decision making

In the first score given immediately after the consultation, but before reviewing the mpMR report, the average score reflecting the anticipated benefit of mpMR was 7 ± 4. The average retrospective review, after reviewing the mpMR report, increased significantly by 20% (*p*<0.0001) to 9 ± 2. For the first set of scores, 65% were ≥ 7/10 while 35% were < 5/10, however for the second set of scores, 78% were ≥7/10 and 6% were < 5/10.

## Discussion

In this study looking at the utility of using MR imaging in AIH we identified four main findings. Firstly, we managed to further the understanding of gradations in biochemical remission by expanding on the concept of deep biochemical remission and identifying the differences between those in deep biochemical remission and those in normal biochemical remission. Secondly, we highlighted the utility of mpMR results in monitoring AIH patients with further insight into patients in biochemical remission with active subclinical disease. Thirdly, we highlighted the potential utility that quantifying the amount of active fibro-inflammatory disease may have on the phenotypic changes that occur in AIH. Lastly, we highlighted the impact mpMR results has on physicians’ intended plans for patient management in a representative real world AIH population.^4^^,^^5^

Serum liver biomarkers are used to monitor disease activity in AIH^4^, ^5^, ^6^ transaminases can be normal (or fluctuate) in patients with chronic hepatitis therefore, normal liver enzymes do not always necessarily indicate the absence of hepatic inflammatory activity.^30^ Although imaging markers correlate significantly with blood markers (as shown by both cT1 and liver stiffness), they also provide additional information. For instance, VCTE liver stiffness,^31^ MRE^32^^,^^33^ and ARFI^34^, ^35^, ^36^ have all shown utility in the characterisation of disease (mainly fibrosis) in AIH.^5^ Similarly, cT1 has shown clinical utility to predict outcomes,^37^^,^^38^ predict flares,^25^ as well as monitor treatment response.^24^ Non-invasive characterisation of disease heterogeneity across the liver is also important in AIH,^1^ and cannot be evaluated by existing imaging or blood tests currently used in clinical practice.^39^ Findings from this study showed that disease heterogeneity (cT1 IQR and pcT1) correlates with blood markers and varies significantly between biochemical remission groups. Thus, understanding the manner in which disease burden across the liver is related to the level of circulating serum markers could potentially be helpful in the clinical assessment of patients with AIH.

Liver biopsy has traditionally been the preferred strategy prior to drug withdrawal, this procedure may not be mandatory in all adults as a similar relapse frequency has been observed between those with or without a pre-withdrawal liver biopsy.^4^^,^^5^^,^^40^ Patients with ALT levels <50% ULN have been shown to have the best outcomes after treatment withdrawal,^5^ with gradations within the normal range of biochemical remission being shown to be predictive of clinical outcomes.^40^ Forty percent of our cohort who were in biochemical remission were classified as being in deep biochemical remission and had significantly less active fibro-inflammatory disease compared to those in normal biochemical remission. Moreover, as both cT1 and pcT1 were significantly higher in those with normal biochemical remission, compared to those in deep biochemical remission, including these markers in the evaluation of patients may provide potentially useful information that can be used to improve disease management. cT1 ≥800 ms in patients with biochemical remission is associated with *a* ≥ 20% chance of developing a flare in AIH^25^ and a cT1>800 ms has been shown to indicate active sub-clinical disease.^29^ Of the 45 patients in normal remission, 16% had evidence of active sub-clinical disease. Thus, 16% of patients with resolved biochemistry in this cohort are potentially at risk of having future disease flares due to persistent fibro-inflammatory activity in the liver that is otherwise undetectable by liver biochemistry.

In this first study assessing the impact of mpMR results on intended clinical management, a 20% increase in physicians’ confidence was observed. Moreover, a decrease of almost 30% (from 35% to 6%) in the lowest ranking scores (scores < 5/10) was also observed. These initial results highlight the potential clinically relevant and disruptive information this technology brings. Therefore, as this study only made use of two clinicians, future multisite studies with a range of clinicians should be performed so as to obtain a better understanding of the impact mpMR results may have on physicians’ patient management.

As this was a real world study, concurrent liver biopsy was not included in the study protocol, as this would have deviated from standard of care. Consequently, we acknowledge the limitations to this study; specifically, the inability to assess relationships between cT1 and histological findings. Nevertheless, previous studies have shown significant correlations between cT1 and histology in both adults^15^^,^^16^^,^^24^ and paediatrics.^29^ This cross sectional study only covered a single time point, thus, following this cohort over time will yield a better understanding of the changes associated with these markers as well as the impact they may have on longitudinal disease monitoring. Lastly, as it has been shown that patient management may vary between clinicians,^7^ future meta-analyses looking at large pooled data across multiple trusts may yield useful information that will support the results presented in this investigation. These studies should also collect data regarding compliance to medication, previous relapses (and their frequency), induction treatment regimens, and other factors which can affect the remission status of a patient. Statistical analyses following these investigations should control for these covariates within any multivariate models generated so as to ensure that any potential confounding effects these factors have will not influence the outcomes observed.

In conclusion, mpMR quantitative biomarkers have shown a positive impact on clinicians intended management plan as well as utility in characterising the fibro-inflammatory status of those in various gradations of biochemical remission. By identifying differences between those in normal biochemical remission, cT1 has shown promise in the phenotyping and risk stratification of individuals with this orphan disease who may not be identified using serum biochemistry and liver stiffness alone. Future analyses investigating the associations between disease and clinical outcomes should also evaluate the utility of markers of disease heterogeneity.

### Data sharing statement

The data and analytic methods used in this study remain the property of the individual study sponsors. All deidentified participant data may be made available to other researchers upon request following permission, investigator support and following a signed data access agreement.

### Funding

This paper presents independent research supported by the Innovate UK grant (104915).

### Contributions

Conceptualisation: MK, AD, RB, MAH

Data collection: MAH, RZA, MNR, MN, LS, ELC

Data curation: RZA, LS, ES

Data analysis: ES

Funding acquisition: MK, AD, RB

Writing original draft: ES

Writing – Review & Editing: ES, MAH, ELC, AD, MK, RB, MN, MNR

All authors reviewed, discussed, and agreed with manuscript.

## Declaration of interests

ES, AD, MK and RB are employees of Perspectum. Perspectum Ltd is a privately funded commercial enterprise that develops medical devices to address unmet clinical needs, including Liver*MultiScan*®. MAH, RZA, MNR, MN, LS and ELC have no conflicts of interest to declare.
